# Volumetric and dosimetric comparison of organs at risk between the prone and supine positions in postoperative radiotherapy for prostate cancer

**DOI:** 10.1186/s13014-018-1023-0

**Published:** 2018-04-17

**Authors:** Subaru Sawayanagi, Hideomi Yamashita, Mami Ogita, Tomoki Kiritoshi, Takahiro Nakamoto, Osamu Abe, Keiichi Nakagawa

**Affiliations:** Departments of Radiology, University of Tokyo Hospital, 7-3-1, Hongo, Bunkyo-ku, Tokyo, 113-8655 Japan

**Keywords:** Prostate cancer, Postoperative radiotherapy, Patient positioning, Belly board, Planning target volume, Small intestine, Overlapping volume, Dose volume histogram

## Abstract

**Background:**

The aim of this study was to evaluate the effects of patient positioning on the volume of organs at risk (OARs) in or near the planning target volume (PTV) and the dose distribution in adjuvant or salvage radiotherapy for prostate cancer after prostatectomy.

**Methods:**

Seventeen patients who received intensity-modulated radiation therapy (66 Gy in 33 fractions) as adjuvant or salvage therapy after prostatectomy were evaluated. All patients underwent CT scans in both the prone (on a belly board) and supine positions. The target volumes and OARs were delineated on each CT series. The planning target volume (PTV) was extended in every direction to generate the PTV + 0.5 cm, PTV + 1 cm, PTV + 2 cm, PTV + 3 cm, and PTV + 4 cm values. The volumes of the OARs overlapping with the PTV and the extended target volumes in the prone and supine position were compared using the Wilcoxon signed-rank test. Dose-volume histogram (DVH) parameters in the prone and supine position were compared using the paired t-test.

**Results:**

The mean overlapping volumes of the small intestine for each of the PTV values were as follows (prone position vs. supine position [mean ± SD]): PTV, 1.5 ± 5.5 cm^3^ vs. 7.9 ± 15.7 cm^3^ (*P* = 0.028); PTV + 0.5 cm, 2.6 ± 8.9 cm^3^ vs. 12.1 ± 22.6 cm^3^ (P = 0.028); PTV + 1 cm, 3.5 ± 11.4 cm^3^ vs. 17.1 ± 29.8 cm^3^ (*P* = 0.028); PTV + 2 cm, 5.6 ± 14.5 cm^3^ vs. 26.8 ± 46.9 cm^3^ (*P* = 0.028); and PTV + 3 cm, 9.0 ± 17.4 cm^3^ vs. 36.5 ± 63.2 cm^3^ (*P* = 0.019), respectively. Some of the overlapping volumes of the rectum and bladder were significantly smaller in the prone position. On the other hand, when the target volume was extended by ≥2 cm, the overlapping volumes of the femurs were significantly larger in the prone position. V15 of the rectum and mean dose and V65 of the bladder were significantly lower in the prone position.

**Conclusions:**

This study indicated that the volumes of the small intestine, rectum, and bladder in or near the PTV decreased when the patient was placed in the prone position on a belly board in postoperative radiotherapy for prostate cancer. The dose distribution seemed superior in the prone position to the supine position.

## Background

It is widely known that adjuvant radiotherapy (RT) after prostatectomy for prostate cancer with adverse pathological findings improves the biochemical progression-free survival, local control, metastasis-free survival, and overall survival [1–5]. In cases in which a patient shows prostate-specific antigen (PSA) relapse after radical prostatectomy, salvage RT also decreases the risk of local recurrence and metastasis and improves prostate cancer-specific survival [6, 7]. Dose escalation contributes to a good prognosis in patients treated with postoperative RT after prostatectomy [8–10]. Although intensity modulated radiation therapy (IMRT) enables us to reduce the risk of radiation-induced toxicity [11, 12], dose escalation is limited by the organs at risk (OARs) surrounding the planning target volume (PTV), particularly the small intestine.

According to a report on uterine cervical cancer, high-dose irradiation is a risk factor for perforation of the small intestine [13]. When treating the pelvic area with RT, it is sometimes necessary to reduce the PTV because the small intestine is located in or near the PTV. A systematic review on the use of an absorbable hydrogel spacer revealed that the placement of a spacer between the prostate and rectum reduced late rectal toxicity and improved the bowel, urinary, and sexual quality of life (QOL) in patients undergoing IMRT for prostate cancer [14]. From this result, the space between the small intestine and the PTV is also assumed to reduce toxicity not only in the small intestine, but in other OARs by making it easier to observe the dose constraints of the other OARs.

Patient positioning may affect the positional relationship among the PTV and the OARs. The patient is sometimes placed in the prone position for RT for pelvic malignancies to reduce the fraction of the small intestine that is exposed to a high dose of radiation. Some studies of three-dimensional conformal radiotherapy (3D-CRT) or IMRT for pelvic malignancies showed that the dose to the small intestine in the prone position was lower than that in the supine position [15, 16], whereas others showed that the dose to the small intestine did not differ to a statistically significant extent between the two patient positions [17, 18]. There have so far been almost no studies about RT in the prone position for prostate cancer in the postoperative setting.

The aim of the present study was to evaluate the effect of patient positioning on the volume of the OARs in or near the PTV and on the dose of the OARs in adjuvant or salvage RT for prostate cancer after prostatectomy.

## Methods

### Patients

Seventeen consecutive patients who received RT after radical prostatectomy as adjuvant or salvage therapy were evaluated. Adjuvant RT was offered to patients with adverse pathologic findings at prostatectomy (e.g., positive surgical margins, seminal vesicle invasion, or extraprostatic extension). Salvage RT was offered to patients with biochemical recurrence, which was defined as an increase in the PSA level until ≥0.2 ng/mL after radical prostatectomy. In some cases, the patients were merely observed and were not treated with adjuvant or salvage RT, even if they had some of the abovementioned conditions; these decisions were mainly based on the judgment of the attending urologists.

### Planning CT

**Fig. 1:**
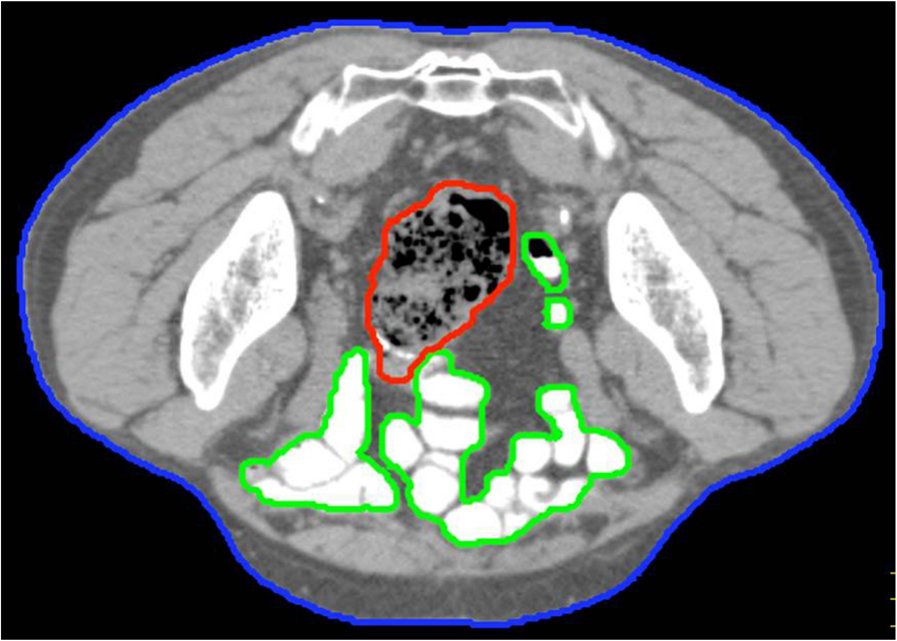
A representative CT image captured after the administration of gastrografin *Blue* skin, *green* small intestine, *red* sigmoid colon

All patients underwent computed tomography (CT) scans reconstructed from 2-mm-thick slices with a full bladder and an empty rectum in both the prone (on a belly board) and supine positions. Patients kept from urinating for over an hour to fill the bladder. If it was difficult to keep from urinating, patients were urged to drink over 300 cm^3^ of water. The goal of bladder filling was the bladder volume more than 150 cm^3^ on the CT images. We administered laxatives to all patients from four days before the planning CT to empty the rectum. From the eighth patient, all patients (*n* = 10, 59% of the patients) took gastrografin orally one hour before the CT scans to enhance their small intestine and differentiate it from the other structures in the body. Figure 1 is a representative CT image captured after the administration of gastrografin.

### Definition of organs at risk and target volumes

We used Pinnacle (Philips, the United States, Andover) as treatment planning systems. The small intestine, rectum, femurs, bladder, and sigmoid colon were delineated as OARs. The anatomical borders of the rectum were the anal verge (caudally), and the region where the rectum turned anterior (cranially). The cranial border of the small intestine was the slice 4 cm above the highest part of the PTV in axial slices. The small intestine, sigmoid colon, and rectum were contoured as loops, including lumens. The caudal border of the femurs was the lowest axial slice including lesser trochanter. The volume of the femurs was defined as the total volume of the bilateral femurs.

**Fig. 2:**
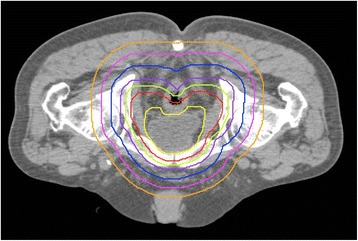
An example of the target volumes and the extended regions of the planning target volume. *Yellow* clinical target volume (CTV), *red* planning target volume (PTV), *green* PTV + 0.5 cm, *purple* PTV + 1 cm, *blue* PTV + 2 cm, *pink* PTV + 3 cm, *orange* PTV + 4 cm

The gross tumor volume (GTV) was not defined because of the postoperative situation. The prostate bed was contoured as the clinical target volume (CTV), according to the Radiation Therapy Oncology Group (RTOG) consensus guidelines [19]. The CTV was extended by 7–8 mm in every direction except posterior with 5 mm extension to generate the PTV. The “PTV + 0.5 cm” was defined as the region extended by 0.5 cm in every direction from the PTV. The PTV + 1 cm, PTV + 2 cm, PTV + 3 cm, and PTV + 4 cm values were also defined as in the case of the PTV + 0.5 cm. Figure 2 shows an example of the target volumes. We calculated the volumes of the OARs overlapping with the PTV and the extended target volumes.

### Traetment planning

Volumetric modulated arc therapy (VMAT) was planned in both the prone and supine positions in all appraisable patients. Some patients whose small intestine was within the PTV in the prone or supine position were regarded as not appraisable and excluded from the comparison of dose distribution in this study because their PTV had to be reduced to protect the small intestine and it should cause unfair comparison, while volumetric analysis of these patients was conducted. A dose of 66 Gy in 33 fractions was prescribed to 95% of the PTV on five days per week. The dose constraints were as follows: D95 (minimum dose received by the highest-dose 95% of the volume) ≥66 Gy of the PTV, V40 (percent volume of the organ receiving 40 Gy radiation) < 60%, V65 < 30%, and V70 < 15% of the bladder, V40 < 60%, V65 < 30%, and V70 < 15% of the rectum, and maximum dose < 50 Gy of the small intestine.

The rectum volume used in the dose constraints was the volume from the slice 1 cm above the highest part of the PTV to the slice 1 cm below the lowest part of the PTV in axial slices. An auto-planning system was used for VMAT planning [20]. Optimization goals of auto-planning were shown in Table 1. We modified the individual plan to satisfy the dose constraints.

**Table 1:** Organs at risk optimization goals of auto-planning

Organ	Dose/volume parameters^a^	Priority
Bladder	V40 < 40%	Medium
	V65 < 20%	Medium
Bladder-PTV^b^	V40 < 40%	High
	V65 < 20%	High
Rectum^c^	V40 < 40%	Medium
	V65 < 20%	Medium
Rectum-PTV^b^	V40 < 40%	High
	V65 < 20%	High
Left femur	Dmax< 45 Gy	High
Right femur	Dmax< 45 Gy	High
Small intestine	Dmax< 48 Gy	High

*Abbreviation: PTV* planning target volume

^a^*Vx* is percent volume of the organ receiving *x* Gy radiation, *Dmax* is maximum dose received by the organ

^b^“*A-PTV*” means the volume of *A* from which the PTV was excluded

^c^Rectum volume here is from the slice 1 cm above the highest part of the PTV to the slice 1 cm below the lowest part of the PTV in axial slices

The distance from the femur to the PTV was measured as the distance from the axial slice including the highest point of the femurs to the axial slice including the highest point of the PTV. Homogeneity index (HI) was defined as a ratio between the maximum dose of the PTV and the minimum dose of the PTV. Conformity index (CI) was defined as a ratio between the volume covered by the minimum dose of the PTV and the PTV.

### Statistical analysis

The overlapping volumes of OARs with the target volumes in the prone and supine position were compared using the Wilcoxon signed-rank test. Dose-volume histogram (DVH) parameters in the prone and supine position were compared using the paired t-test. The correlation of age, body mass index (BMI), the interval between surgery and the start of RT, bladder volume, CI, and the distance from femur to the PTV with the maximum dose of the small intestine were evaluated using Pearson’s correlation coefficient. *P* values of < 0.05 were considered to indicate statistical significance. All statistical analyses were performed using the R software program (The R Foundation for Statistical Computing, Austria, Vienna).

## Results

Patients received planning CT scans from March 2017 through November 2017. The patient characteristics are summarized in Table 2. The median age was 71 years (range, 67–84). The median (BMI) was 23.5 (20.3–26.7). The median time between prostatectomy and the initiation of RT was 24.9 (2–188) months. Seven patients (41.2% of the patients) received adjuvant RT and ten patients (58.8%) received salvage RT.

**Table 2:** Patient characteristics

Variable	(*N* = 17)
	*n* (%)
Age, median (range) in years	71 (67–84)
Body mass index, median (range)	23.5 (20.3–26.7)
Gleason score	
≤7	7(41.2%)
≥8	10 (58.8%)
Resection margins	
R0	7 (41.2%)
R1	10 (58.8%)
Extracapsular invasion	
No	6 (35.3%)
Yes	11 (64.7%)
Seminal vesicle invasion	
No	8 (47.1%)
Yes	9 (52.9%)
Lymphadenectomy performed	
No	3 (17.6%)
Yes	14 (82.4%)
Lymph node classification	
N0	16 (94.1%)
N1	1 (5.9%)
Adjuvant or salvage	
Adjuvant	7 (41.2%)
Salvage	10 (58.8%)
Interval between surgery and RT start, median (range) in months	24.9 (2.0–188.0)
Treatment position	
Prone	15 (88.2%)
Supine	2 (11.8%)
ADT during RT	
No	9 (47.1%)
Yes	8 (52.9%)

*Abbreviations RT* radiotherapy, *VMAT* volumetric modulated arc therapy, *ADT* androgen deprivation therapy

In four patients (23.5%), the small intestine overlapped with the PTV in the supine position, but no part of the small intestine was included in the PTV in the prone position. Although the small intestine and the PTV overlapped in both the prone and the supine position in two patients (11.8%), the overlapping volumes were reduced in the prone position in both patients. Dosimetric comparison was conducted in 11 patients (64.7%), whose small intestine was not within the PTV in both the prone and supine positions. All 17 patients were included in volumetric analysis. The mean doses of the periphery of the PTV, PTV + 0.5 cm, PTV + 1 cm, PTV + 2 cm, PTV + 3 cm, and PTV + 4 cm were 63 Gy, 48 Gy, 34 Gy, 23Gy, 16 Gy, and 12 Gy, respectively.

**Fig. 3:**
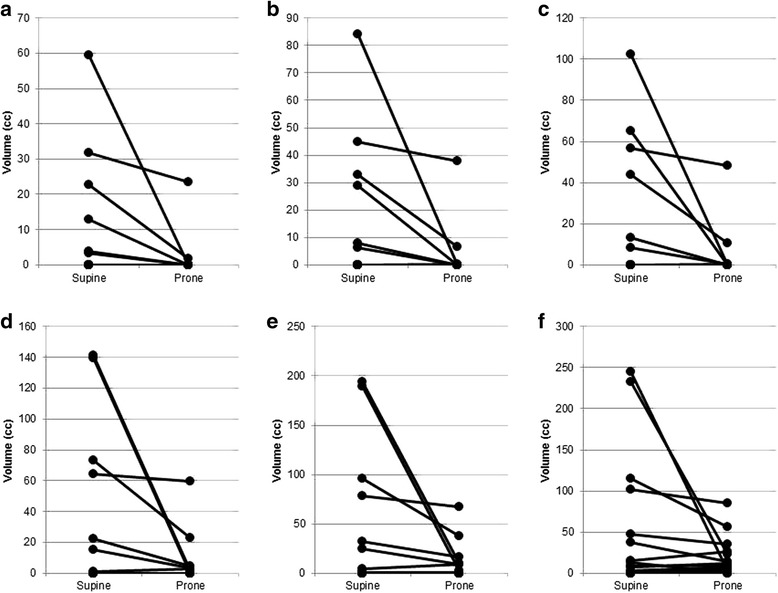
Overlapping volumes of the PTV and the extended volumes with the small intestine. **a** PTV ∩ small intestine (*P* = 0.036). **b** PTV + 0.5 cm ∩ small intestine (*P* = 0.035). **c** PTV + 1 cm ∩ small intestine (*P* = 0.035). **d** PTV + 2 cm ∩ small intestine (*P* = 0.035). **e** PTV + 3 cm ∩ small intestine (*P* = 0.021). **f** PTV + 4 cm ∩ small intestine (*P* = 0.16).* Abbreviations: PTV* planning target volume, *∩* overlapping. The plots of the same patients are connected by black lines. Overlapping volumes were zero in both the prone and supine position in eleven patients of the PTV, ten patients of the PTV + 0.5 cm, PTV + 1 cm, and PTV + 2 cm, five patients of the PTV + 3 cm, and two patients of the PTV + 4 cm. Their lines and plots are overlapping at the bottom of each graph. *P* values were calculated by the Wilcoxon signed-rank test

In volumetric analysis in the 17 patients, the overlapping volumes of the OARs are shown in Table 3. The overlapping volumes of the small intestine were as follows (prone position [mean ± SD] vs. supine position, respectively): PTV, 1.5 ± 5.5 cm^3^ vs. 7.9 ± 15.7 cm^3^ (*P* = 0.036); PTV + 0.5 cm, 2.6 ± 8.9 cm^3^ vs. 12.1 ± 22.6 cm^3^ (*P* = 0.035); PTV + 1 cm, 3.5 ± 11.4 cm^3^ vs. 17.1 ± 29.8 cm^3^ (*P* = 0.035); PTV + 2 cm, 5.6 ± 14.5 cm^3^ vs. 26.8 ± 46.9 cm^3^ (*P* = 0.035); and PTV + 3 cm, 9.0 ± 17.4 cm^3^ vs. 36.5 ± 63.2 cm^3^ (*P* = 0.021). The overlapping volumes of the small intestine in the prone and supine positions are shown in Fig. 3. The small intestine was more than 1 cm away from the PTV in 12 patients (70.6%) in the prone position and 10 patients (58.8%) in the supine position.

**Table 3:** Overlapping volumes of OARs with the PTV and the extended regions

	Prone		Supine		
	mean (cc)	SD (cc)	mean (cc)	SD (cc)	*P* value^a^
Small intestine					
PTV	1.5	5.5	7.9	15.7	0.036^†^
PTV + 0.5 cm	2.6	8.9	12.1	22.6	0.035^†^
PTV + 1 cm	3.5	11.4	17.1	29.8	0.035^†^
PTV + 2 cm	5.6	14.5	26.8	46.9	0.035^†^
PTV + 3 cm	9.0	17.4	36.5	63.2	0.021^†^
PTV + 4 cm	16.5	22.6	48.8	77.3	0.16
Rectum					
PTV	10.9	4.2	12.4	4.4	0.064
PTV + 0.5 cm	25.2	7.4	29.2	8.0	0.0093^†^
PTV + 1 cm	38.6	10.6	44.7	11.1	0.0056^†^
PTV + 2 cm	60.3	17.2	67.4	20.4	0.064
PTV + 3 cm	71.2	21.4	76.5	27.6	0.38
PTV + 4 cm	74.5	22.3	80.1	32.1	0.61
Bilateral femurs^b^					
PTV	0.0	0.0	0.0	0.0	
PTV + 0.5 cm	0.1	0.3	0.1	0.2	0.34
PTV + 1 cm	1.6	1.9	1.1	1.6	0.12
PTV + 2 cm	14.5	9.1	11.9	8.7	0.0093^†^
PTV + 3 cm	42.4	14.8	37.5	15.3	0.0021^†^
PTV + 4 cm	77.0	16.3	71.2	17.8	0.0039^†^
Bladder					
PTV	62.7	18.7	78.4	22.4	0.0021^†^
PTV + 0.5 cm	87.9	24.0	102.9	25.3	0.0032^†^
PTV + 1 cm	110.2	30.2	122.8	28.2	0.0032^†^
PTV + 2 cm	147.7	45.3	153.2	40.1	0.017^†^
PTV + 3 cm	176.5	62.2	174.8	56.5	0.064
PTV + 4 cm	197.3	78.9	190.1	72.8	0.089
Sigmoid colon					
PTV	1.5	2.0	2.6	4.3	0.12
PTV + 0.5 cm	4.1	5.4	5.9	8.5	0.26
PTV + 1 cm	7.1	8.9	9.6	12.6	0.12
PTV + 2 cm	16.1	15.7	20.2	19.6	0.27
PTV + 3 cm	28.6	20.6	33.4	24.1	0.59
PTV + 4 cm	42.9	25.1	45.6	28.5	0.71

*Abbreviation****,*** *OARs* organs at risk, *PTV* planning target volume, *SD* standard deviation

^a^by Wilcoxon signed-rank test

^b^The total volume of the bilateral femurs

†*P* < 0.05

The overlapping volumes of the rectum with PTV + 0.5 cm and PTV + 1 cm, and those of the bladder with PTV, PTV + 0.5 cm, PTV + 1 cm, and PTV + 2 cm were significantly smaller in the prone position. The overlapping volumes of the rectum and bladder are shown in Table 3. The overlapping volumes of the femurs with PTV + 2 cm (14.5 ± 9.1 cm^3^ vs. 11.9 ± 8.7 cm^3^, *P* = 0.0093), PTV + 3 cm (42.4 ± 14.8 cm^3^ vs. 37.5 ± 15.3 cm^3^, *P* = 0.0021), and PTV + 4 cm (77.0 ± 16.3 cm^3^ vs. 71.2 ± 17.8 cm^3^, *P* = 0.0039) were significantly larger in the prone position (Table 3).

**Fig. 4:**
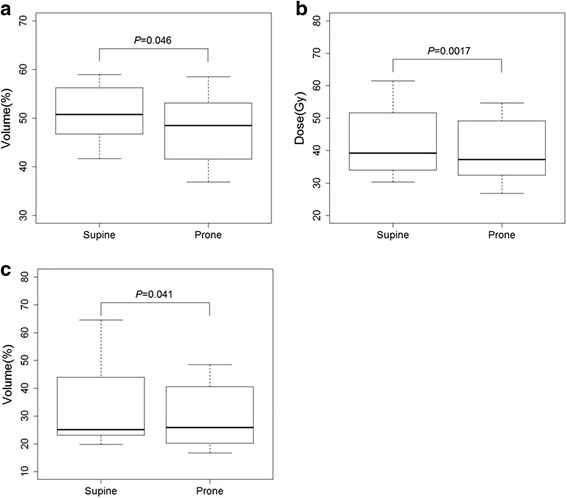
Comparison of DVH parameters between the supine and prone positions. **a** V40 of the rectum. **b** Mean dose of the bladder. **c** V65 of the bladder. *Abbreviations: DVH* dose-volume histogram, *Vx* percent volume of the organ receiving *x* Gy radiation. *P* values were calculated by the paired t-test

In dosimetric comparison in the 11 appraisable patients, V40 of the rectum (47.9 ± 7.3% vs. 50.7 ± 5.8%, *P* = 0.046), mean dose of the bladder (39.5 ± 9.7 Gy vs. 42.0 ± 10.2 Gy, *P* = 0.0017), and V65 of the bladder (30.1 ± 11.7% vs. 33.5 ± 14.1%, *P* = 0.041) were significantly lower in the prone position (Fig. 4).

DVH parameters of the small intestine were as follows (prone position [mean ± SD] vs. supine position, respectively): maximum dose, 11.8 ± 11.8 Gy vs. 9.0 ± 9.3 Gy (*P* = 0.068); V15, 0.9 ± 2.6 cm^3^ vs. 0.4 ± 1.2 cm^3^ (*P* = 0.28). No part of the small intestine received over 45 Gy in the 11 patients.

DVH parameters of the rectum were as follows (prone position [mean ± SD] vs. supine position, respectively): mean dose, 42.9 ± 2.6 Gy vs. 43.5 ± 1.8 Gy (*P* = 0.21); maximum dose, 71.0 ± 0.6 Gy vs. 71.2 ± 0.5 Gy (*P* = 0.66); V70, 0.2 ± 0.2% vs. 0.3 ± 0.2% (*P* = 0.28); V65, 14.5 ± 2.6% vs. 14.8 ± 2.9% (*P* = 0.72); V50, 34.7 ± 6.0% vs. 36.9 ± 5.0% (*P* = 0.053).

DVH parameters of the bladder were as follows (prone position [mean ± SD] vs. supine position, respectively): maximum dose, 71.0 ± 0.7 Gy vs. 71.1 ± 0.4 Gy (*P* = 0.54); V70, 0.4 ± 0.7% vs. 0.3 ± 0.3% (*P* = 0.59); V50, 42.1 ± 14.2% vs. 45.6 ± 16.8% (*P* = 0.074); V40, 48.9 ± 15.2% vs. 52.3 ± 17.8% (*P* = 0.067).

DVH parameters of the sigmoid colon were as follows (prone position [mean ± SD] vs. supine position, respectively): maximum dose, 38.5 ± 28.1 Gy vs. 48.0 ± 24.9 Gy (*P* = 0.13); V65, 0.7 ± 1.3 cm^3^ vs. 1.3 ± 3.0 cm^3^ (*P* = 0.38); V50, 1.8 ± 3.2 cm^3^ vs. 2.9 ± 5.8 cm^3^ (*P* = 0.30); V40, 2.5 ± 4.3 cm^3^ vs. 4.0 ± 7.5 cm^3^ (*P* = 0.28).

DVH parameters of the femurs were as follows (prone position [mean ± SD] vs. supine position, respectively): maximum dose, 42.7 ± 5.1 Gy vs. 43.5 ± 2.0 Gy (*P* = 0.63); V40, 0.8 ± 0.9 cm^3^ vs. 1.0 ± 1.1 cm^3^ (*P* = 0.59); V30, 32.4 ± 17.5 cm^3^ vs. 41.7 ± 14.7 cm^3^ (*P* = 0.043). No part of the femurs received over 50 Gy in the 11 patients.

DVH parameters of the PTV were as follows (prone position [mean ± SD] vs. supine position, respectively): V107% (percent volume of the target volume receiving 107% of the prescribed dose), 0.1 ± 0.2% vs. 0.1 ± 0.1% (*P* = 0.77); HI, 1.23 ± 0.03 vs. 1.25 ± 0.04 (*P* = 0.21); CI, 1.43 ± 0.10 vs. 1.43 ± 0.11 (*P* = 0.97).

Among the 11 patients, contoured small intestine was enhanced by gastrografin in five patients. We first conducted VMAT planning of these five patients without replacement of Hounsfield unit (HU) of the small intestine. We secondly substituted HU of water for HU of the small intestine and recalculated DVHs using the same beam as the plan without replacement. We compared DVHs of the plan without replacement and the one with replacement using the paired t-test to investigate the effect of the enhancement of small intestine on the dose distribution. V105% of the PTV was 6.0 ± 4.8 [mean ± SD] % without replacement and 6.0 ± 4.8% with replacement (*P* = 0.17). V100%, V95%, V90%, and V85% of the PTV did not change with or without replacement in all five patients.

**Fig. 5:**
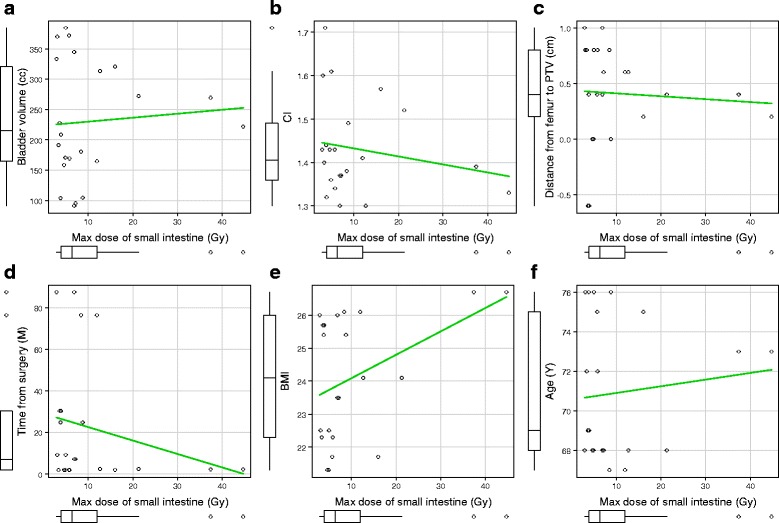
Correlation between clinical parameters and maximum dose of the small intestine. **a** Bladder volume (**b**) CI (**c**) The distance from the femur to the PTV (**d**) Time from surgery to the start of RT (**e**) BMI (**f**) Age. *Abbreviations****:*** *CI* conformity index, *PTV* planning target volume, *RT* radiotherapy, *BMI* body mass index. Green lines mean regression lines

Bladder volume (*r* = 0.074; 95% CI, − 0.359 to 0.480; *P* = 0.74), CI (*r* = − 0.185; 95% CI, − 0.563 to 0.257; *P* = 0.41), the distance from the femur to the PTV (*r* = − 0.063; 95% CI, − 0.472 to 0.368; *P* = 0.78), time from surgery to the start of RT (*r* = − 0.234; 95% CI, − 0.597 to 0.208; *P* = 0.29), BMI (*r* = 0.409; 95% CI, − 0.015 to 0.709; *P* = 0.058), and age (*r* = 0.107; 95% CI, − 0.330 to 0.506; *P* = 0.64) did not have correlation with maximum dose of the small intestine (Fig. 5).

## Discussion

The small intestine is sometimes a dose-limiting organ in RT for pelvic malignancies. Yamashita et al. [13] reported seven patients who suffered from small bowel perforation after intracavitary radiation therapy in combination with external beam radiation therapy for uterine cervical cancer. In six of these patients the biological effective doses around the site of bowel perforation were 101.0–437.7 Gy (the dose distribution data was unavailable for the remaining patient) [13].

In patients with pelvic malignancies undergoing 3D-CRT or IMRT, the dose of the small intestine in the prone position has been shown to be lower than that in the supine position [15]. The same study also showed that the dose reduction was greater when the patient was in the prone position using a belly board. In a dosimetric study that investigated 10 rectal cancer patients who underwent IMRT [16], the prone position reduced the dose to the small bowel, while the dose distribution of the PTV was almost the same as that in the supine position. Another study of 3D-CRT for high-risk localized or locally advanced prostate cancer [17] showed that in the supine position, the volumes of the femoral heads and sigmoid colon that received a high dose of radiation were smaller than those in the prone position and no significant difference was found in the small intestine. In a recent study of the use of VMAT in the preoperative treatment of rectal cancer [18], the dose to the small intestine did not differ to a statistically significant extent between the prone and supine orientations; however, the setup reproducibility in the supine position was higher than that in the prone position. To the best of our knowledge, the present study is the first to show the effect of treatment positioning on the orientation of the OARs and the dose distribution during RT for prostate cancer in the postoperative setting.

In six patients (35.3%), the PTV included a fraction of the small intestine when they were in the supine position. They needed to be treated in the prone position in order to keep the small intestine away from the PTV. Even though the small intestine was more than 1 cm away from the PTV in the supine position in 10 patients (58.8%), the prone position seemed to be better because there was more space between the PTV and the small intestine, which enabled us to give priority to reducing the radiation dose that the other OARs would receive as well as the homogeneity of the dose distribution in the PTV. Actually, even though DVHs of the small intestine were not significantly different between in the prone position and in the supine position, the doses of the rectum and the bladder were lower in the prone position.

The femurs were nearer to the PTV when the patient was in the prone position, but DVHs of the femurs in the prone position was not inferior to those in the supine position. Although we administered oral gastrografin before CT scans, the enhanced regions of the small intestine did not affect the dose distribution.

The present study was associated with some limitations. In particular, we extended the CTV in the prone position by the same extent as the supine position, but the setup error in the prone position may bigger than that in the supine position. Even though we did not collect the data about the reproducibility of RT, we used a belly board and the patients underwent CT scans before every RT to compare them to the planning CT and correct the patient position if needed, which would contribute to improving the reproducibility in the prone position.

## Conclusions

Our findings suggest that the volumes of the small intestine, rectum, and bladder in or near the PTV in adjuvant or salvage radiotherapy for prostate cancer after prostatectomy decreased when the patient was placed in the prone position on a belly board. DVHs of the rectum and the bladder were better in the prone position than in the supine position.

## Abbreviations

3D-CRT: Three-dimensional conformal radiotherapy
BMI: Body mass index
CT: Computed tomography
CTV: Clinical target volume
DVH: Dose-volume histogram
GTV: Gross tumor volume
HI: Homogeneity index
HU: Hounsfield unit
IMRT: Intensity modulated radiation therapy
OARs: Organs at risk
PSA: Prostate-specific antigen
PTV: Planning target volume
QOL: Quality of life
RT: Radiotherapy
RTOG: Radiation Therapy Oncology Group
VMAT: Volumetric modulated arc therapy
